# An integrated cofactor and co-substrate recycling pathway for the biosynthesis of 1,5-pentanediol

**DOI:** 10.1186/s12934-024-02408-y

**Published:** 2024-05-06

**Authors:** Wenfeng Hua, Bo Liang, Suhui Zhou, Qiushui Zhang, Shuang Xu, Kequan Chen, Xin Wang

**Affiliations:** Nanjing Tech University, Nanjing, China

**Keywords:** 1,5-Pentanediol, *Escherichia coli*, Cofactor and co-substrate recycling system, Transamination

## Abstract

**Background:**

1,5-pentanediol (1,5-PDO) is a linear diol with an odd number of methylene groups, which is an important raw material for polyurethane production. In recent years, the chemical methods have been predominantly employed for synthesizing 1,5-PDO. However, with the increasing emphasis on environmentally friendly production, it has been a growing interest in the biosynthesis of 1,5-PDO. Due to the limited availability of only three reported feasible biosynthesis pathways, we developed a new biosynthetic pathway to form a cell factory in *Escherichia coli* to produce 1,5-PDO.

**Results:**

In this study, we reported an artificial pathway for the synthesis of 1,5-PDO from lysine with an integrated cofactor and co-substrate recycling and also evaluated its feasibility in *E.coli*. To get through the pathway, we first screened aminotransferases originated from different organisms to identify the enzyme that could successfully transfer two amines from cadaverine, and thus GabT from *E. coli* was characterized. It was then cascaded with lysine decarboxylase and alcohol dehydrogenase from *E. coli* to achieve the whole-cell production of 1,5-PDO from lysine. To improve the whole-cell activity for 1,5-PDO production, we employed a protein scaffold of EutM for GabT assembly and glutamate dehydrogenase was also validated for the recycling of NADPH and α-ketoglutaric acid (α-KG). After optimizing the cultivation and bioconversion conditions, the titer of 1,5-PDO reached 4.03 mM.

**Conclusion:**

We established a novel pathway for 1,5-PDO production through two consecutive transamination reaction from cadaverine, and also integrated cofactor and co-substrate recycling system, which provided an alternative option for the biosynthesis of 1,5-PDO.

**Supplementary Information:**

The online version contains supplementary material available at 10.1186/s12934-024-02408-y.

## Introduction

1,5-pentanediol (1,5-PDO) is an aliphatic diol with odd carbon atoms [1]. It is an important raw material for synthesizing polyesters, unsaturated polyesters, and polyurethanes [2], and can also be used in the production of plastics, cosmetics, and food. Currently, 1,5-PDO is mainly produced through the chemical process using dimethylglutarate as a raw material [3]. With biomass-derived materials of furfural and tetrahydrofurfuryl alcohol, the production of 1,5-PDO was also achieved by a chemical catalysis process [3, 4]. However, these processes exhibited some issues such as high cost, cumbersome steps, and environmental safety [5]. With increasing concerns on green and sustainable production, developping the biological process for 1,5-PDO production was highly desired.

Due to the absence of a natural metabolic pathway for 1,5-PDO biosynthesis, it has attracted a great interest in designing artificial pathways for 1,5-PDO biosynthesis [5]. Until now on, only three lysine-derived artificial pathways have been successfully implemented to produce 1,5-PDO in *E. coli*. In 2020, Wang et al. designed an artificial pathway derived from lysine with six steps, and established it in *E. coli* [6]. Lysine was first converted to 5-aminovalerate (5-AVA) using lysine monooxygenase (DavB) and 5-aminopentamidase (DavA), and 5-AVA was subsequently converted to 5-hydroxyvalerate (5-HV) through the aminotransferase and aldosterone reductase. Carboxylate reductase and alcohol dehydrogenase finally reduced 5-HV to 1,5-PDO. Cen et al*.* reported an 1,5-PDO pathway from lysine by employing seven enzymes of DavB, DavA, aminotransferase, aldosterone reductase, CoA-transferase, CoA-acylated acetaldehyde dehydrogenase, and alcohol dehydrogenase [5]. Based on previous research, Cen et al. reported an efficient cadaverine-based pathway to produce 1,5-PDO through seven steps. lysine decarboxylase (CadA), cadaverine aminotransferase (PatA), glutaraldehyde dehydrogenase (PatD), aminotransferase, carboxylate reductase and alcohol dehydrogenase [7]. The further development of artificial 1,5-PDO biosynthetic pathway is benefit for expanding new methods for its bioproduction.

Here, we envisaged the development of a novel biosynthetic pathway for 1,5-PDO synthesis from lysine via the cadaverine with the following important objectives: (1) simple and direct catalysis of amino group to hydroxyl group with minimal steps; (2) a redox self-sufficient network with high efficiency in the use of atoms; (3) using cheap substrate and avoiding the formation of any by-product. Based on these principles, we redesigned a smart pathway through a three-step reaction starting from lysine. In the pathway, lysine was first converted to cadaverine through CadA, which was further converted to the intermediate glutaraldehyde through the action of aminotransferase with α-KG as an amino receptor. Finally, 1,5-PDO was produced through the action of alcohol dehydrogenase. The high efficient conversion of lysine to cadaverine by lysine decarboxylase of CadA has been studies in many works [8–10], while alcohol dehydrogenase of YahK was extensively employed in alcohol biosynthesis due to its high activity in reducing aldehyde groups to hydroxyl groups [11, 12]. PatA has been identified as converting cadaverine to 5-aminopentanal for the aminotransferase [13]. However, the sequential transfer of two amino groups from cadaverine to glutaraldehyde by aminotransferases has not been reported yet. In the study by Tatiana et al., the aminotransferase of SAV2585 was used to convert adipic aldehyde into adipic diamine, demonstrating the potential of using a single aminotransferase to transfer two amines [14]. It is still a challenge to identify aminotransferases that could convert cadaverine to glutaraldehyde for accessing our designed pathway. Additionally, it is vital to achieve cofactor and co-substrate recycling during the biocatalysis process due to the high cost of cofactors in enzymatic reactions [11, 15]. Therefore, we need to consider the glutamate and the supply of NADPH required for alcohol dehydrogenase in our pathway.

Overall, we have developed a new pathway for the biosynthesis of 1,5-PDO from lysine, and an integrated NADPH and glutamate recycling were also performed by adding GdhA. To enable the pathway function, we initially screened seven aminotransferases from various organisms and successfully identified GabT to transfer two amines from cadaverine. We then employed a protein scaffold of EutM for GabT assembly to enhance the whole-cell activity for 1,5-PDO production and the cofactor and co-substrate recycling system was subsequently characterized. Following it, the cultivation and bioconversion conditions were optimized to further improve the pathway efficiency for 1,5-PDO production.

## Methods

### Plasmid construction

The plasmids used in this study were listed in Table 1. Vectors of pTrc99a and pRSFDuet were used for gene manipulations and protein expression. The *CadA* gene was amplified by PCR from *E.coli* MG1655 and cloned into pRSFDuet using restriction enzymes EcoRI and SacI, then *GabT* was also amplified by PCR from *E.coli* MG1655 and cloned into pRSFDuet using NdeI and BglII, resulting in plasmid pRSFDuet-*CadA-GabT*. For construction of plasmids pTrc99a-*YahK*, the *YahK* gene was amplified by PCR from *E.coli* MG1655 and inserted fragments between NcoI and KpnI. *E.coli* MG1655 was used as a template for PCR amplification of *GdhA* and inserted into XbaI and HindIII of pTrc99a-*YahK* to obtain pTrc99a-*YahK-GdhA*. The *GabT* ~ *Spytag* and *EutM* ~ *SpyCatcher* were obtained by fragment fusion using linker fragments. pRSFDuet-*CadA-GabT* ~ *Spytag-EutM* ~ *SpyCatcher* was obtained by replacing the GabT fragment in pRSFDuet-CadA-GabT with GabT ~ Spytag and inserting the fragment EutM ~ SpyCatcher into KpnI.

**Table 1:** Strains and plasmids used in this study

Strains or plasmids	Description	References
*Strains*		
BL21(DE3)	Used as host strain	Invitrogen
BL21-GY	BL21(DE3) harboring plasmid pRSFDuet-*CadA-GabT* and pTrc99a-*YahK*	This study
BL21-SC-GY	BL21(DE3) harboring plasmid pRSFDuet-*CadA-GabT* ~ *Spytag* and pTrc99a-*EutM-YahK*	This study
BL21-SC-GY-*GdhA*	BL21(DE3) harboring plasmid pRSFDuet-*CadA-GabT* ~ *Spytag* and pTrc99a-*EutM-YahK-GdhA*	This study
*Plasmids*		
pRSFDuet	expression vector, Km^R^, P_T7_, ori	This study
ppTrc99a	expression vector, Am^R^, P_Trc_, ori	This study
pET28a	expression vector, Km^R^, P_T7_, ori	
pRSFDuet-*CadA-GabT*	Km^R^, pRSFDuet harboring *CadA* and *GabT*	This study
pTrc99a*-YahK*	Am^R^, pTrc99a harboring *YahK*	This study
pRSFDuet-*CadA-GabT* ~ *Spytag-EutM* ~ *SpyCatcher*	Km^R^, pRSFDuet harboring *CadA, GabT, Spytag, EutM* and* SpyCatcher*	This study
pTrc99a*-YahK-GdhA*	Am^R^, pTrc99a harboring *YahK and GdhA*	This study
pET28a-*SpuC*	Km^R^, pET28a harboring *SpuC*	This study
pET28a-*QLH*	Km^R^, pET28a harboring *QLH*	This study
pET28a-*Spo3471*	Km^R^, pET28a harboring *Spo3471*	This study
pET28a-*PatA*	Km^R^, pET28a harboring *PatA*	This study
pET28a-*SAV2585*	Km^R^, pET28a harboring *SAV2585*	This study
pET28a-*GabT*	Km^R^, pET28a harboring *GabT*	This study
pET28a-*SM5064*	Km^R^, pET28a harboring *SM5064*	This study

### Strains and media

The strains used in this study were listed in Table 1. *E. coli* BL21 (DE3) was used as host cells. The enzymes used in this work were searched from the NCBI database and summarized in Additional file 1: Table S1. The specific primer sequence was shown in Additional file 1: Table S2. The strains were cultured in Luria Bertani medium (LB) at 37°C and shaking at 200 rpm. The LB medium consisted of 10 g/L tryptone, 5 g/L yeast extract, and 5 g/L sodium chloride.

### Expression and validation of aminotransferases

To compare the efficiency of different aminotransferases in the transamination of cadaverine, the plasmids of each aminotransferase were expressed in *E. coli* BL21(DE3), and plated on the corresponding resistant plates to obtain single colonies. The correct colonies were selected, and then cultured at 37°C for 8–12 h in 5 mL of LB liquid medium with *Kan*. The bacterial suspension was transferred to 100 mL of LB liquid medium. When OD_600_ was about 0.8, 0.5 mM IPTG was added. After induction at 18°C for 16–20 h, the cells were collected by centrifugation at 4 °C and 6000 rpm, and then washed three times with PBS salt buffer solution. The whole cells were lysed by sonication. Subsequently, precipitation and supernatant were obtained by centrifugation. Finally, the expression was verified by sodium dodecyl sulfate polyacrylamide gel electrophoresis (SDS-PAGE).

### Determination of optimal aminotransferase for the whole-cell catalysis

To determine the optimal aminotransferase for the production of 1,5-PDO in vivo, their activity of sequential transfer of two amino groups from cadaverine to glutaraldehyde was first evaluated by the whole cell bioconversion experiment. The cell pellets containing aminotransferase were resuspended by using PBS buffer, and added into the reaction mixture containing 20 mM cadaverine, 20 mM α-KG, 20 mM glucose and1 mM PLP. Glutaraldehyde production was assayed to determine the optimal aminotransferase in the whole-cell catalytic production of 1,5-PDO. The cascade reaction of aminotransferase and alcohol dehydrogenase (YahK) were performed under the same conditions, and 1,5-PDO production was measured to re-verify the optimal aminotransferase in vivo.

### Whole cell catalytic production of 1,5-PDO

To verify the whole cell catalytic production of 1,5-PDO, the co-expressed bacteria BL21-GY, BL21-SC-GY, and BL21-SC-GY-*GdhA* were activated and coated on the corresponding resistant plates to obtain single colonies. The single colonies were selected and cultured at 37°C for 8–12 h in 5 mL of LB liquid medium with corresponding antibiotics. The bacterial solution was transferred to 100 mL of LB liquid medium with resistance for further culture, When OD_600_ was about 0.6 ~ 0.8, IPTG with a final concentration of 0.5 mM was added, and the bacteria were collected after induction at 18°C for 18–20 h. Then, the cells were collected by centrifugation at 4°C, 6000 rpm and 15 min, washed with PBS salt buffer solution and centrifugated for later use.

Cells were resuspended by PBS for whole-cell catalytic reaction, the OD_600_ of which was 40. The catalytic reaction substrates for BL21-GYand BL21-SC-GY were 20 mM lysine, 20 mM α-KG, 20 mM glucose and 1 mM PLP. BL21-SC-GY-*GdhA* replaced α-KG with the same concentration of glutamate and removed glucose. The reaction system was placed at 220 rpm at 37°C for 12 h. The whole cell catalyst for the control group was designed as empty bacterial BL21 (DE3).

### Optimization of cell cultivation conditions

After obtaining the recombinant strain for 1,5-PDO production, the cell cultivation conditions were subsequently optimized for the high whole-cell activity. To achieve it, the induction time, IPTG addition amount, and culture time were optimized. When cell grew into OD_600_ of 0.2, 0.4, 0.6, 0.8 and 1.0, the induction was carried out at different cell concentrations, respectively. The effects of IPTG concentration (0.25, 0.5, 1 and 1.5 mM) were also evaluated. Finally, we investigated the effects of cultivation time ranging from 14 to 20 h. The whole-cell ability was comparatively analyzed by measuring the yield of 1,5-PDO, where the whole cells with the highest activity under each condition was defined as 100%.

### Optimization of bioconversion conditions

The bioconversion conditions to produce 1,5-PDO, including reaction temperature, whole-cell concentration, and substrate ratio were also optimized. The effect of different catalytic temperatures on the production of 1,5-PDO was investigated when the temperatures were set at 20°C, 25°C, 30°C, 35°C, and 40°C, respectively. Secondly, gradient comparison was conducted on the catalytic cell concentration within the range of OD_600_ of 10–50 to determine the optimal cell concentration. Finally, we investigated the effects of the ratio of lysine to glutamate (2:1, 1:1, 1:2, 1:3) in the catalytic reaction on the production of 1,5-PDO. The whole-cell ability was comparatively analyzed by measuring the yield of 1,5-PDO, where the whole cells with the highest activity under each condition was defined as 100%.

### Quantification of L-lysine, glutaraldehyde and 1,5-PDO

The initial and final concentrations of L-lysine were measured by SBA-40E biosensor. The analysis of glutaraldehyde and 1,5-PDO were performed by Agilent 1290 HPLC instrument and Aminex HPX-87H column (300 × 7.88 mm) chromatography column. The column temperature was kept constant at 60°C and 20 µL were injected into the mobile phase consisting of 8 mM H_2_SO_4_. The flow rate was set to 0.6 mL/min.

## Result

### 5-PDO synthesis pathway catalyzed by cascade of multiple enzymes

Organisms do not possess natural biosynthetic pathways to produce 1,5-PDO. Previous studies have reported three lysine-derived artificial pathways for 1,5-PDO bioproduction, which were successfully established in *E. coli* [5–7]. A recent study systematically compared eight potential pathways for converting lysine into 1,5-PDO and reported that a cadaverine-based pathway has an advantage in producing 1,5-PDO due to its high theoretical yield [7]. The pathway comprised two modules. Module 1 of the cadaverine-based 5-hydroxyvalerate synthesis included lysine decarboxylase, putrescine aminotransferase, aldehyde dehydrogenase, 4-aminobutyrate aminotransferase, and alcohol dehydrogenase, which converted lysine to 5-hydroxyvalerate. Module 2 of the 1,5-PDO synthesis module comprised carboxylic acid reductase and alcohol dehydrogenase. A total of seven enzymes were required, along with two molecules of NADPH, ATP, and α-KG. It was hypothesized that if the aminotransferase could directly catalyze the two amino groups of cadaverine to glutaraldehyde, lysine could be converted directly to 1,5-PDO in only three steps: decarboxylation, transamination, and reduction. Thus, we designed a pathway from lysine to 1,5-PDO using three enzymes: lysine decarboxylase, aminotransferase, and alcohol dehydrogenase. The pathway consumed two molecules of NADPH and α-KG and produced two molecules of glutamate, which did not require ATP. The pathway in our study was expected to lead to a simpler biosynthetic route for 1,5-PDO synthesis, in which aminotransferase was identified as the key step.

**Fig. 1:**
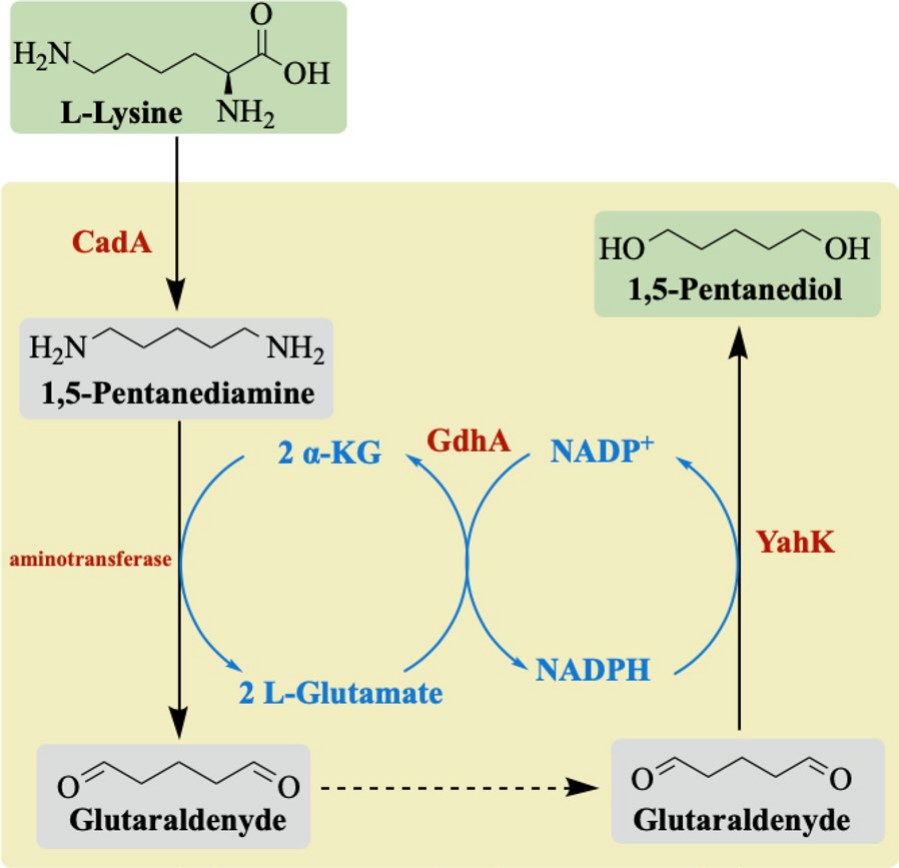
An artificial pathways for the biosynthesis of 1,5-PDO from lysine. The black lines represented the reaction process, the red lines represented the key reaction steps, and the blue lines represented the co-factors involved in the production process. Abbreviations: CadA, lysine decarboxylase (EC 4.1.1.18); YahK, alcohol dehydrogenase (EC 1.1.1.1); GdhA, glutamate dehydrogenase (EC 1.4.1.4)

To improve the economic efficiency of the pathway, it would be highly desirable to incorporate the efficient recycling of expensive cofactors and avoid the generation of extra byproduct. In the transamination reaction towards cadaverine, aminotransferase typically employed α-KG as the primary cofactor, which acted as an amine acceptor. However, the high cost of α-KG was a significant drawback in the designed pathway [16]. Furthermore, NADPH was necessary as a cofactor for alcohol dehydrogenase to convert aldehyde to alcohol, which reduced the economic feasibility of the pathway. Additionally, the reversible reaction of α-KG to glutamate, catalyzed by aminotransferase, has gained attention as an effective cofactor regeneration system [17]. A previous study confirmed that transamination and dehydrogenation processes provided additional driving forces, which formed a self-sufficient substrate circulation system to promote the progression of reactions for better [18]. The production of α-KG from glutamate could be achieved by L-glutamate oxidase (GOX), L-glutamate dehydrogenase, or L-amino acid oxidase [19, 20]. The study conducted by *Jinbin L. *et al*.* successfully constructed a biocatalytic oxidation–reduction cascade reaction to produce α-phenylethanol. The reaction consisted of aminotransferase, aldosterone reductase, and glutamate dehydrogenase. Glutamate dehydrogenase catalyzed the NADP^+^-dependent oxidative deamination of glutamate to produce α-KG and NADPH [15]. Here, we employed glutamate dehydrogenase to establish a cofactor and co-substrate recycling system (Fig. 1).

### Identification of aminotransferases capable of transferring two amino groups from cadaverine to glutaraldehyde for 1,5-PDO production

**Fig. 2:**
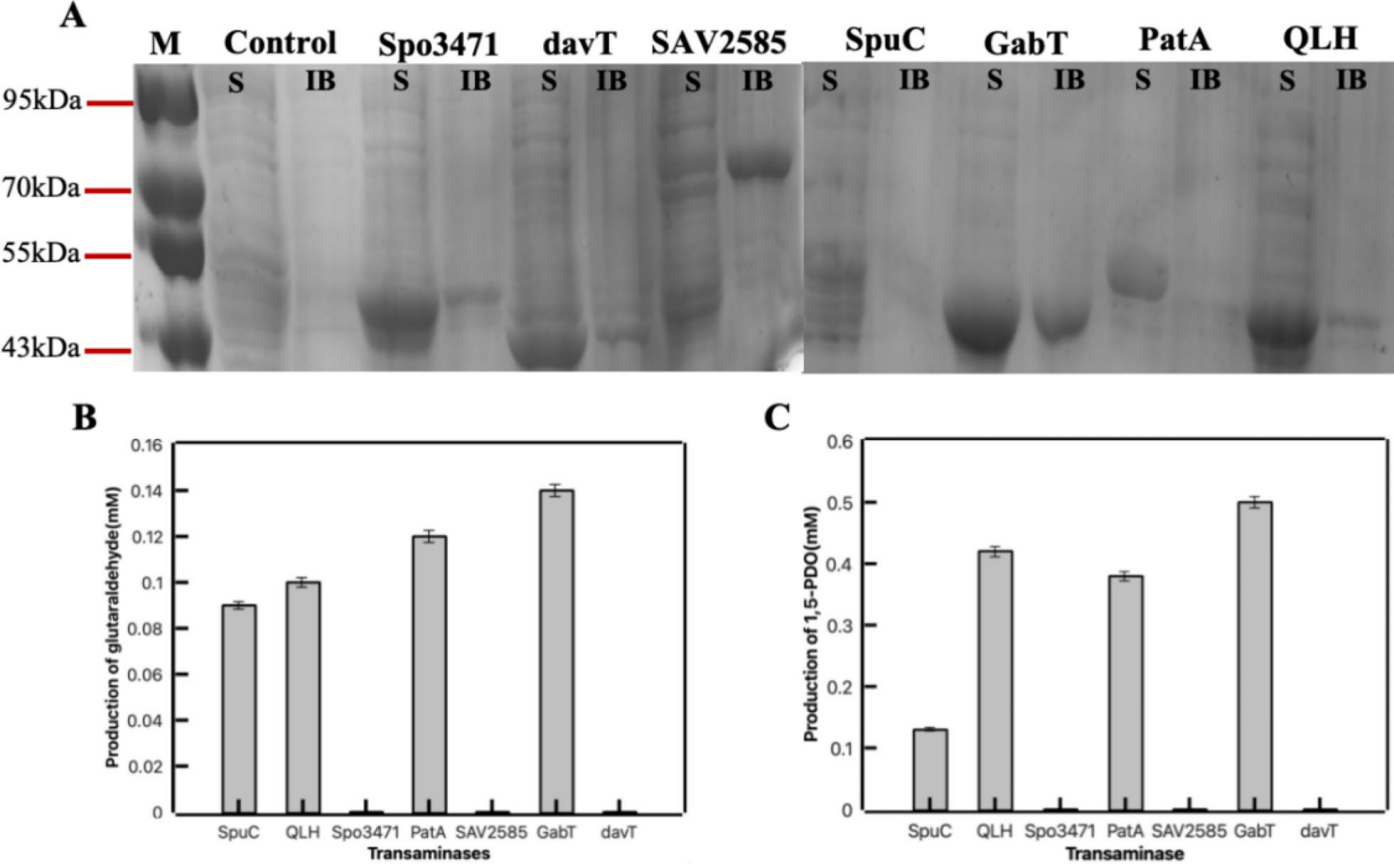
The identification of aminotransferase for 1,5-PDO production in vivo. **A** Protein expression of different aminotransferases in *E. coli*. Control, *E. coli* BL21(DE3) containing empty plasmid; Spo3471, *E. coli* BL21(DE3) expressing aspartate aminotransferase from *Ruegeria pomeroyi* (EC2.6.1.1); davT, *E. coli* BL21(DE3) expressing 5-aminovalerate aminotransferase from *Pseudomonas* (EC2.6.1.48); *E. coli* BL21(DE3) expressing SAV2585, adenosylmethionine-8-amino-7-oxononanoate aminotransferase from *Streptomyces avermitilis* (EC2.6.1.62); SpuC, *E. coli* BL21(DE3) expressing putrescine-pyruvate aminotransferase from *Pseudomonas aeruginosa* (EC2.6.1.113); GabT, *E. coli* BL21(DE3) expressing 4-aminobutyrate-2-oxoglutarate aminotransferase from *E. coli* (EC2.6.1.19); PatA, *E. coli* BL21(DE3) expressing putrescine-2-oxoglutarate aminotransferase from *E. coli* (EC2.6.1.82); QLH, *E. coli* BL21(DE3) expressing aspartate-prephenate aminotransferase from *Paracoccus denitrificans* (EC2.6.1.8). **B** Glutaraldehyde production from cadaverine by cells expressing different aminotransferases. **C** 1,5-PDO production by cells expressing different aminotransferases in cascade with YahK

In order to construct the artificial biosynthetic pathway for 1,5-PDO synthesis, we first identified an aminotransferase enzyme capable of catalyzing the conversion of cadaverine into glutaraldehyde. Here, we tested seven aminotransferases originating from different organisms, including SpuC from *Pseudomonas aeruginosa*, QLH from *Paracoccus denitrificans*, Spo3471 from *Ruegeria pomeroyi*, PatA from *E. coli*, SAV2585 from *Streptomyces avermitilis*, GabT from *E. coli,* and DavT from *Pseudomonas*. All corresponding genes were synthesized with optimized codons, ligated into the plasmid of pET28a, and transformed into *E. coli* BL21(DE3). SDS-PAGE analysis was carried out to verify the expression of seven aminotransferases in *E. coli*. As shown in Fig. 2A, soluble protein expression could be observed for all seven aminotransferases. The molecular weights of SpuC, QLH, Spo3471, PatA, SAV2585, GabT, and DavT were 53.7 kDa, 51.3 kDa, 52.9 kDa, 57.8 kDa, 66 kDa, 45.8 kDa, and 44.8 kDa, respectively.

Then, a bioconversion experiment was conducted to determine the optimal aminotransferase for 1,5-PDO production in vivo. The cells expressing different aminotransferases were used as the biocatalyst in the reaction mixture containing 100 mM PBS buffer (pH 8.0), 20 mM cadaverine, 20 mM α-KG and 1 mM PLP. After a reaction of 12 h, the generation of glutaraldehyde was determined by HPLC analysis. The whole-cells harboring SpuC, QLH, PatA, and GabT enzymes all exhibited the ability in catalyzing the transformation of cadaverine into glutaraldehyde as shown in Fig. 2B. Notably, the whole-cells expressing GabT manifested the highest yield of glutaraldehyde production.

We then co-expressed alcohol dehydrogenase (YahK) in the cells that contained aminotransferase to identify aminotransferase candidates for the synthesis of 1,5-PDO. The cells were incubated in PBS buffer containing 20 mM cadaverine, 20 mM α-KG and 1 mM PLP. The results presented in Fig. 2C, alongside The simplified HPLC diagram depicted in Additional file 1: Figure S1, corroborated the potential of aminotransferases SpuC, QLH, PatA, and GabT for the production of 1,5-PDO in our designed pathway. Among the evaluated enzymes, the whole-cells expressing GabT emerged as the most efficacious, evidenced by the detection of 0.5 mM 1,5-PDO. These results also demonstrated the feasibility of the short route we developed to produce 1,5-PDO from lysine using only three enzymes.

### Immobilization of aminotransferase by a self-assembling protein scaffold system to improve its catalytic efficiency

Enzyme immobilization is commonly employed in biotransformation, which has the potential to improve enzyme stability and reaction efficiency [21]. Because the ammonia conversion reaction was a rate-limiting step in the generation of 1,5-PDO, we employed a self-assembling protein scaffold system to encapsulate GabT in *E. coli* and assessed its effect on the production of 1,5-PDO. In the study by Guoqiang et al., the ethanolamine utilization (Eut) bacterial microdomain (BMC) of *Salmonella enteric* was a special protein nanostructure. Its outer shell protein of EutM could also self-assemble in vivo as large protein filaments [22]. What’s more, EutM protein scaffold can be easily heterologous expressed in *E. coli*. It has been developed as a convenient platform for immobilization of enzymes in the biocatalytic system [23]. A previous study successfully assembled an alcohol dehydrogenase into EutM scaffold, which exhibited better activity and stability [24]. Similarly, in the study by Xianhan et al*.*, they used the EutM protein scaffold to co-immobilize α-amylase and trehalose synthase, maintaining enzyme activity for approximately three times higher than free enzymes [25]. In summary, it proved that EutM protein scaffold could be a feasible way to improve the production of 1,5-PDO.

**Fig. 3:**
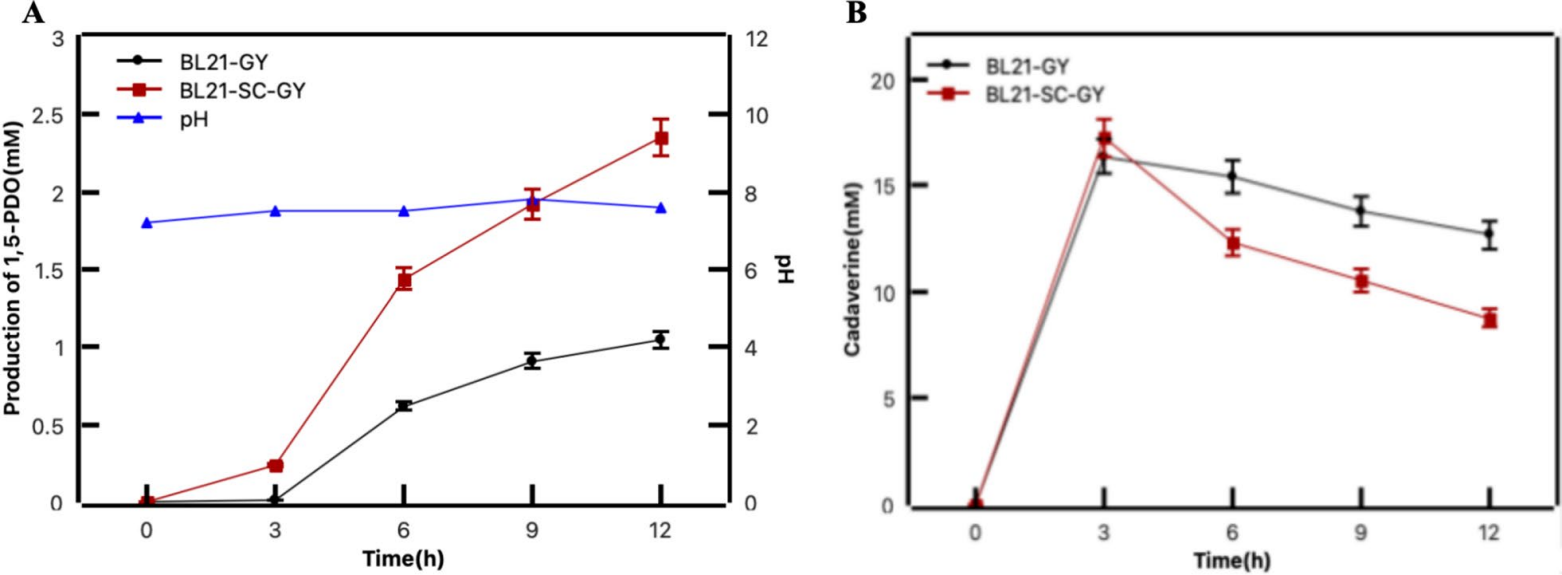
The immobilization of GabT by a self-assembling protein scaffold EutM for improved 1,5-PDO production. **A** Production of 1,5-PDO from lysine by the strain of BL21-SC-GY and BL21-GY. **B** The change of intermediate cadaverine during the 1,5-PDO production in the strain of BL21-SC-GY and BL21-GY. BL21-GY, 1,5-PDO original production strain; BL21-SC-GY, 1,5-PDO production strain after addition of scaffolds

To integrate the aminotransferase GabT into the EutM scaffold, a genetic fusion strategy was employed whereby the SpyCatcher domain was appended to EutM, and concurrently, the SpyTag domain was attached to GabT. The two fused proteins were co-expressed with CadA and YahK in *E. coli* to obtain BL21-SC-GY, which was subsequently assessed for its catalytic proficiency to produce 1,5-PDO. The strain of BL21-SC-GY was incubated in PBS buffer containing 20 mM lysine, 20 mM α-KG and 1 mM PLP. We compared the efficiency of 1,5-PDO production before and after the assembly. Notably, as delineated in Fig. 3A, the immobilization of GabT onto the EutM scaffold significantly enhanced the production of 1,5-PDO, exhibiting a 2.5-fold increase compared to the control strain after 12 h. Meanwhile, the accumulation of the intermediate of cadaverine was also observed to be reduced in the strain of BL21-SC-GY (Fig. 3B). These results clearly demonstrated that the assembly of aminotransferase GabT into the protein scaffold was conducive to the continuous transamination reaction.

### An integrated cofactor and co-substrate recycling by incorporating glutamate dehydrogenase

**Fig. 4:**
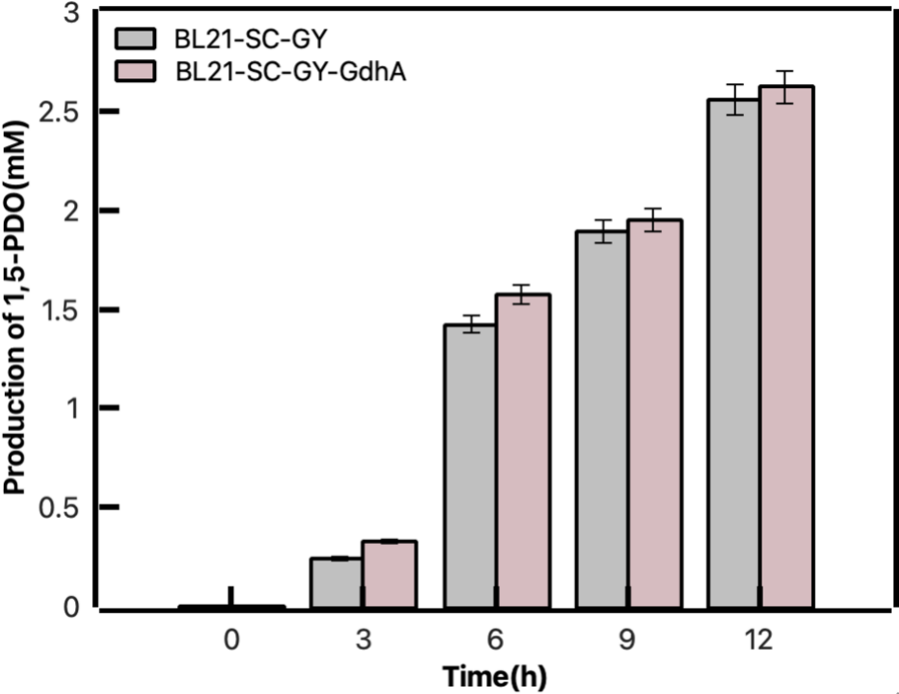
Comparison of catalytic production of 1,5-PDO before and after addition of GdhA

The dependence on the expensive cofactors could increase the cost of the biocatalysis process. In our designed pathway, considering the high cost of NADPH and α-KG, an auxiliary enzyme of glutamate dehydrogenase(GdhA) was employed to achieve redox-cofactor recycling, as well as the co-substrate regeneration. Specifically, glutamate dehydrogenase catalyzed the NADP^+^-dependent oxidative deamination of L-glutamate to α-KG. During the biosynthesis of α-phenylethanol, the co-substrate and redox equivalents were regenerated simultaneously by glutamate dehydrogenase [15]. Also, it has been previously reported that it could enhance 1,5-PDO production by the introduction of glucose dehydrogenase (GDH) for NADPH regeneration [26]. Similarly, as shown in Fig. 4, the catalytic system for BL21-SC-GY was 20 mM lysine, 20 mM α-KG, 20 mM glucose and 1 mM PLP. The catalytic system for BL21-SC-GY-*GdhA* was 20 mM lysine, 20 mM glutamate and 1 mM PLP. When we co-expressed GdhA with GabT and YahK, supplemented glutamate instead of α-KG and removed glucose, the production of 1,5-PDO was not decreased. These results suggested that GdhA not only regenerated the co-substrate of α-KG from glutamate in the transamination reaction, but also provided cofactor NADPH for alcohol dehydrogenase. It indicated that it was successful for implementation of integrated cofactor and co-substrate recycling in our pathway.

### Optimization of cultivation conditions to to enhance the ability of recombinant cells

**Fig. 5:**
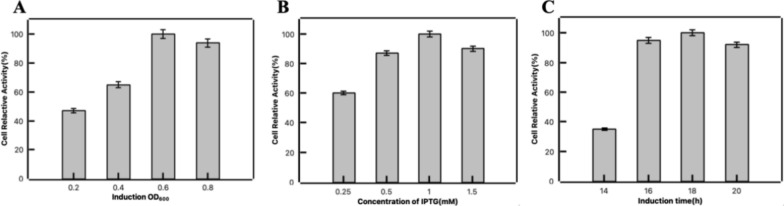
Optimization of culture conditions for obtaining optimal cell viability. **A** The effect of induction OD_600_ on cell activity. **B** The effect of IPTG concentration of on cell activity. **C** The effect of cultivation time on cell activity

As catalysts for multi-enzyme cascade catalytic reactions, cells need to maintain a relatively high level of activity for the efficient 1,5-PDO production. Therefore, we investigated the effects of cell concentration, inducer concentration and induction time to obtain the cells with optimal viability to increase the yield of 1,5-PDO. Cell ability was characterized by the yield of 1,5-PDO. The highest activity under each condition was defined as 100% and differences between cells were obtained by comparing relative ability. Firstly, we examined the effect of cell concentration on cell viability during induction. The inducer IPTG was added when the cells reached an OD_600_ of 0.2, 0.4, 0.6, or 0.8. Whole-cell catalysis experiments were performed using 20 mM lysine, 20 mM glutamate and 1 mM PLP as substrates. Figure 5A showed that the relative cell ability was highest at an OD_600_ of 0.6. The effect of inducer concentration on cell catalysis was then investigated by adding different concentrations of IPTG. The results in Fig. 5B showed that the relative cell ability was highest at the IPTG concentration of 1 mM. Finally, the effect of induction cultivation time on cell ability was examined, and the relative cell ability reached the highest level after 18 h of cultivation. As the induction cultivation time increased, cell ability decreased significantly.

### Effect of bioconversion conditions on 1,5-PDO synthesis

**Fig. 6:**
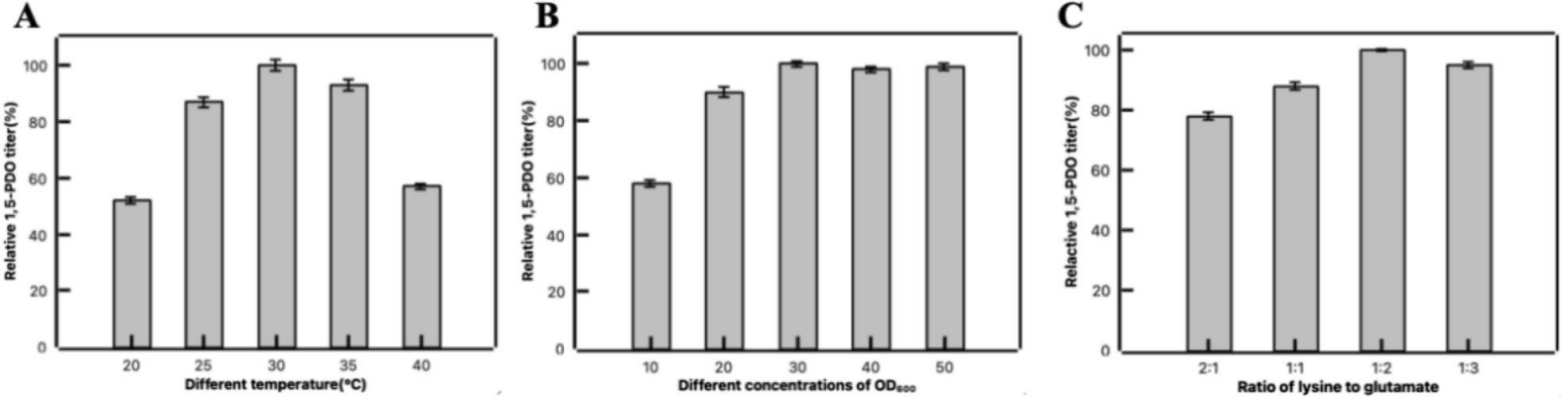
Optimizing catalytic conditions to improve 1,5-PDO production efficiency. **A** The effect of reaction temperature on 1,5-PDO production. **B** The effect of cell concentration on 1,5-PDO production. **C** The effect of different substrate concentration ratios on 1,5-PDO production

The bioconversion conditions were further optimized to increase the final yield of 1,5-PDO. The effects of different temperatures on the whole-cell catalytic production of 1,5-PDO were investigated. As shown in Fig. 6A, the titer of 1,5-PDO increased as the catalytic temperature increased from 18°C to 30°C, reaching its highest level at 30°C. Additionally, the concentration of the whole-cells was found to have an impact on the final production of 1,5-PDO. We conducted a gradient experiment to increase the concentration of catalytic cells from 10 to 50 in OD_600_. Whole-cell catalysis experiments were performed using 20 mM lysine, 20 mM glutamate and 1 mM PLP as substrates. Figure 6B showed that as the cell concentration increased from 10 to 30, the production of 1,5-PDO also increased. However, the catalytic reaction tended towards equilibrium when OD_600_ increased from 30 to 50. Previous reports have suggested that the concentration of amino receptors can influence the transamination reaction. Thus, we optimized the ratio of substrate lysine to glutamate for catalytic production. Figure 6C showed that the highest production efficiency of 1,5-PDO was achieved when the molar ratio of lysine to glutamate was 1:2.

**Fig. 7:**
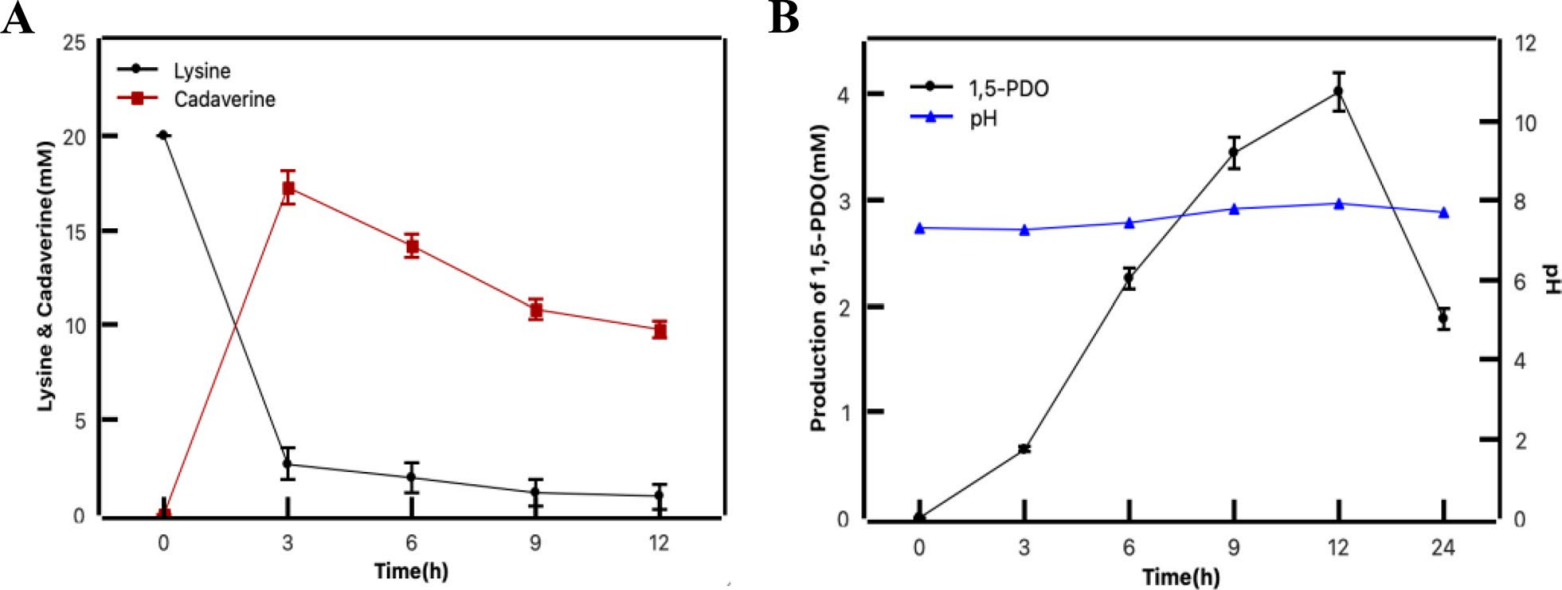
Whole-cell catalytic production of 1,5-PDO in optimal conditions. **A** The change of lysine and cadaverine during the 1,5-PDO production in the strain of BL21-SC-GY under optimal conditions. **B** Production of 1,5-PDO from lysine by the strain of BL21-SC-GY under optimal conditions

To investigate the optimal catalytic system for 1,5-PDO production, we conducted an experiment using a reaction mixture containing a cell concentration (OD_600_) of 30 and a substrate with a lysine to glutamate molar ratio of 1:2. The changes in whole-cell catalysis of the substrate lysine and the intermediate cadaverine were shown in Fig. 7A. Figure 7B showed that after 12 h, the highest level of 1,5-PDO production was achieved, resulting in 4.03 mM (0.42 g/L) of 1,5-PDO. However, a precipitous decline in 1,5-PDO yield was observed upon extending the catalytic reaction duration to 24 h, which is suspected that the presence of reductases in *E. coli* degraded polyols. Previous studies have mentioned that alcohol dehydrogenase was a bifunctional enzyme for catalyzing the interconversion between aldehydes and alcohols. Therefore, endogenous genes in *E. coli* can be screened to identify and knock out 1,5-PDO degrading genes.

## Discussion

1,5-PDO as an important chemical intermediate plays an indispensable role in industries such as polyester and coatings, which has a broad range of downstream applications and promising market prospects. However, only three feasible pathways for producing 1,5-PDO in *E. coli* have been reported [5–7], so it is necessary to develop and expand new pathways for biosynthesis of 1,5-PDO. In this study, we proposed a novel pathway for synthesizing 1,5-PDO, which was composed of lysine decarboxylase, aminotransferase, and alcohol dehydrogenase. The EutM protein scaffold was used to assemble the aminotransferase, which improved the whole-cell catalytic efficiency towards 1,5-PDO synthesis. Furthermore, the integration of GdhA into the system established a mechanism for the recycling of cofactor and co-substrate, thereby presenting a viable strategy for the reduction of production costs. We then optimized the cultivation conditions to obtain highly viable cells as biocatalysts to further enhance 1,5-PDO production. Bioconversion conditions were also optimized to result in the production of 4.03 mM of 1,5-PDO after 12 h.

The selection of aminotransferases was one of the most important considerations in our pathway. A previous study showed that aminotransferase SAV2685 could achieve two amino transfers (Tatiana et al. 2020) [14], while it was not suitable for the amino transfer of cadaverine in our pathway. Suitable aminotransferases can be identified by using cadaverine as a substrate to detect the production of glutaraldehyde in bioconversion. The optimal aminotransferase was then cascaded with alcohol dehydrogenase for catalysis, and was verified by the production of 1,5-PDO. Ultimately, GabT was identified as the aminotransferase in the pathway and further study for it was still required. In a previous study, it was reported that deleting the N-terminal His-tag can enhance the activity of GabT (Lijun et al*.* 2019) [27]. Since we chose pRSFDuet for plasmid construction which had its own His-tag, we could improve enzyme activity by removing it in the future. In addition, another study suggested that modifying key residues adjacent to the substrate binding pockets of aminotransferases could potentially improve their activity (Li-Qun et al. 2022) [28].

Glutamate dehydrogenase exists in all living organisms, which is often used to reduce glutamate to α-KG (Hong et al. 2017) [29]. In a previous study, glutamate dehydrogenase was classified into NAD^+^ and NADP^+^ dependent types to provide different cofactors required for biological reactions (Katsuhiko et al. 2018) [30]. We selected NADP^+^-dependent glutamate dehydrogenase because NADPH was necessary for alcohol dehydrogenase to convert glutaraldehyde to 1,5-PDO. A previous study found that the introduction of NADP^+^ dependent glutamate dehydrogenase improved the synthesis of alcohols by enhancing the circulation of cofactor and co-substrate. It not only reduced the cost of substrate usage but also solved the problem of byproduct accumulation caused by ammonia conversion (Jinbin et al*.* 2022). [31]. However, as a bidirectional enzyme, glutamate dehydrogenase may not be as effective in supplying α-KG. Therefore, glutamate oxidase (GOX) was introduced, which greatly reduced the demand for α-KG in other study [16]. Meanwhile, the addition of GOX and the use of monosodium glutamate as a substitute for α-KG in whole-cell catalysis were also shown to be a viable approach to substrate cycling(Haeng-Geun et al. 2022) [17].

In general, the biosynthesis of 1,5-PDO can be achieved through the decarboxylation, transamination, and reduction pathway using lysine as a substrate. A cofactor and substrate recycling system was established to enhance the synthesis of 1,5-PDO. After condition optimization, 4.02 mM of 1,5-PDO was obtained. However, the production of 1,5-PDO was found to be constrained by various factors. Notably, the enzymatic activity of aminotransferases emerged as a critical limiting factor, which could be improved through protein modification and other means as mentioned in previous studies. Although the integration of GabT with the EutM scaffold was pursued to enhance transamination efficiency, the resultant yield of 1,5-PDO did not meet anticipated levels, which suggested that EutM was not the most suitable protein scaffold for transamination. Based on it, different combinations of scaffolds could be explored to improve 1,5-PDO yield. Furthermore, despite efforts to refine cell cultivation and bioconversion conditions, the yields remained suboptimal, which indicated that further optimization, such as adjusting the induction temperature during cell culture, could be instrumental in enhancing cell viability and production efficiency consequently. An additional consideration is the osmotic effect and damage to the cells in whole-cell catalysis. Different surfactants can be added to the catalysis to improve the efficiency of the movement of substances in and out of the cells. Meanwhile, the introduction of glutamate oxidase into the catalytic process may offer further optimization avenues. Concurrently, the identification and knockout of endogenous alcohol dehydrogenases in *E. coli* that degrade 1,5-PDO can prevent product loss. To produce 1,5-PDO more economically, the fermentation of glucose to generate 1,5-PDO will be explored. *E. coli* NT1003, identified in previous studies for its high lysine production during growth with glucose as substrate, can be selected as the engineering strain for fermentation. Therefore, we can introduce the production pathway into *E. coli* NT1003 to obtain higher titers of 1,5-PDO.

### Supplementary Information

**Additional file 1: Figure S1**. HPLC simplified diagram of 1,5-PDO standard and the involvement of GabT in whole-cell catalytic production of 1,5-PDO. **Table S1.** The gene sequences used in this study. **Table S2.** Primers used in this study.

## Data Availability

All data generated or analyzed during this study are included in this article.
