# Supplementary material for: An integrated cofactor and co-substrate recycling pathway for the biosynthesis of 1,5-pentanediol
Source: Microb Cell Fact. 2024 May 6;23:132. doi: 10.1186/s12934-024-02408-y (PMC11075332; doi:10.1186/s12934-024-02408-y)
Supplement: Supplementary file 1 — Additional file 1: Figure S1. HPLC simplified diagram of 1,5-PDO standard and the involvement of GabT in whole-cell catalytic production of 1,5-PDO. Table S1. The gene sequences used in this study. Table S2. Primers used in this study. [file 12934_2024_2408_MOESM1_ESM.docx]

# Additional file for

# An intergrated Cofactor and Co-substrate Recycling pathway for the Biosynthesis of 1,5-pentanediol

Wenfeng Hua^†^, Bo Liang^†^, Suhui Zhou^†^, Qiushui Zhang^†^, Shuang Xu^†^, Kequan Chen^†^, Xin Wang^† *^

Email: [xinwang1988@njtech.edu.cn](mailto:xinwang1988@njtech.edu.cn)

**This files include:**

Figures S1 to S2

Table S1 to S2


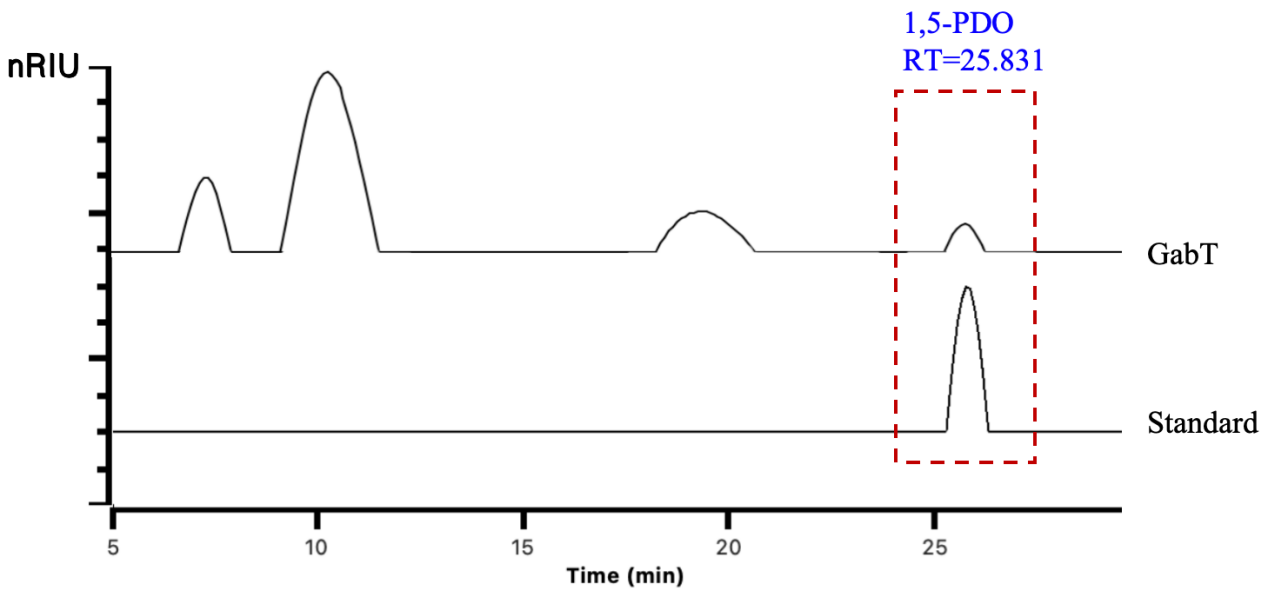


**Figure S1. HPLC simplified diagram of 1,5-PDO standard and the involvement of GabT in whole-cell catalytic production of 1,5-PDO**

**Table S1.** The gene sequences used in this study

| **Genes** | **Sequences (5´-3´)** |
| --- | --- |
| **CadA** | ATGAACGTTATTGCAATATTGAATCACATGGGGGTTTATTTTAAAGAAGAACCCATCCGTGAACTTCATCGCGCGCTTGAACGTCTGAACTTCCAGATTGTTTACCCGAACGACCGTGACGACTTATTAAAACTGATCGAAAACAATGCGCGTCTGTGCGGCGTTATTTTTGACTGGGATAAATATAATCTCGAGCTGTGCGAAGAAATTAGCAAAATGAACGAGAACCTGCCGTTGTACGCGTTCGCTAATACGTATTCCACTCTCGATGTAAGCCTGAATGACCTGCGTTTACAGATTAGCTTCTTTGAATATGCGCTGGGTGCTGCTGAAGATATTGCTAATAAGATCAAGCAGACCACTGACGAATATATCAACACTATTCTGCCTCCGCTGACTAAAGCACTGTTTAAATATGTTCGTGAAGGTAAATATACTTTCTGTACTCCTGGTCACATGGGCGGTACTGCATTCCAGAAAAGCCCGGTAGGTAGCCTGTTCTATGATTTCTTTGGTCCGAATACCATGAAATCTGATATTTCCATTTCAGTATCTGAACTGGGTTCTCTGCTGGATCACAGTGGTCCACACAAAGAAGCAGAACAGTATATCGCTCGCGTCTTTAACGCAGACCGCAGCTACATGGTGACCAACGGTACTTCCACTGCGAACAAAATTGTTGGTATGTACTCTGCTCCGGCAGGCAGCACCATTCTGATTGACCGTAACTGCCACAAATCGCTGACCCACCTGATGATGATGAGCGATGTTACGCCAATCTATTTCCGCCCGACCCGTAACGCTTACGGTATTCTTGGTGGTATCCCACAGAGTGAATTCCAGCACGCTACCATTGCTAAGCGCGTGAAAGAAACACCAAACGCAACCTGGCCGGTACATGCTGTAATTACCAACTCTACCTATGATGGTCTGCTGTACAACACCGACTTCATCAAGAAAACACTGGATGTGAAATCCATCCACTTTGACTCCGCGTGGGTGCCTTACACCAACTTCTCACCGATTTACGAAGGTAAATGCGGTATGAGCGGTGGCCGTGTAGAAGGGAAAGTGATTTACGAAACCCAGTCCACTCACAAACTGCTGGCGGCGTTCTCTCAGGCTTCCATGATCCACGTTAAAGGTGACGTAAACGAAGAAACCTTTAACGAAGCCTACATGATGCACACCACCACTTCTCCGCACTACGGTATCGTGGCGTCCACTGAAACCGCTGCGGCGATGATGAAGGGTAATGCTGGTAAGCGTCTGATCAACGGTTCCATTGAACGTGCGATCAAATTCCGTAAAGAGATCAAACGTCTGAGAACGGAATCTGATGGCTGGTTCTTTGATGTTTGGCAGCCGGATCATATCGATACGACTGAATGCTGGCCGCTGCGTTCTGACAGCACCTGGCACGGCTTCAAAAACATCGATAACGAGCACATGTATCTTGACCCGATCAAAGTCACCCTGCTGACTCCGGGGATGGAAAAAGACGGCACCATGAGCGACTTTGGTATTCCGGCCAGCATCGTGGCGAAATACCTCGACGAACATGGCATCGTTGTTGAGAAAACCGGTCCGTATAACCTGCTGTTCCTGTTCAGCATCGGTATCGATAAGACCAAAGCACTGAGCCTGCTGCGTGCTCTGACTGACTTCAAACGTGCGTTCGACCTGAACCTGCGTGTGAAAAACATGCTGCCGTCTCTGTATCGTGAAGATCCTGAATTCTATGAAAACATGCGTATTCAGGAACTGGCTCAAAATATCCACAAACTGATTGTTCACCACAATCTGCCGGATCTGATGTATCGCGCATTTGAAGTGCTGCCGACGATGGTAATGACTCCGTATGCTGCGTTCCAGAAAGAGCTGCACGGTATGACCGAAGAAGTTTACCTCGACGAAATGGTAGGTCGTATTAACGCCAATATGATCCTTCCGTATCCGCCGGGAGTTCCTCTGGTAATGCCGGGTGAAATGATCACCGAAGAAAGCCGTCCGGTTCTGGAGTTCCTGCAGATGCTGTGTGAAATCGGCGCTCACTATCCGGGCTTTGAAACCGATATTCACGGTGCATACCGTCAGGCTGATGGCCGCTATACCGTTAAGGTATTTAA |
| **GabT** | ATGAACAGCAATAAAGAGTTAATGCAGCGCCGCAGTCAGGCGATTCCCCGTGGCGTTGGGCAAATTCACCCGATTTTCGCTGACCGCGCGGAAAACTGCCGGGTGTGGGACGTTGAAGGCCGTGAGTATCTTGATTTCGCGGGCGGGATTGCGGTGCTCAATACCGGGCACCTGCATCCGAAGGTGGTGGCCGCGGTGGAAGCGCAGTTGAAAAAACTGTCGCACACCTGCTTCCAGGTGCTGGCTTACGAGCCGTATCTGGAGCTGTGCGAGATTATGAATCAGAAGGTGCCGGGCGATTTCGCCAAGAAAACGCTGCTGGTTACGACCGGTTCCGAAGCGGTGGAAAACGCGGTAAAAATCGCCCGCGCCGCCACCAAACGTAGCGGCACCATCGCTTTTAGCGGCGCGTATCACGGGCGCACGCATTACACGCTGGCGCTGACCGGCAAGGTGAATCCGTACTCTGCGGGCATGGGGCTGATGCCGGGTCATGTTTATCGCGCGCTTTATCCTTGCCCGCTGCACGGCATAAGCGAGGATGACGCTATCGCCAGCATCCACCGGATCTTCAAAAATGATGCCGCGCCGGAAGATATCGCCGCCATCGTGATTGAGCCGGTTCAGGGCGAAGGCGGTTTCTACGCCTCGTCGCCAGCCTTTATGCAGCGTTTACGCGCTCTGTGTGACGAGCACGGGATCATGCTGATTGCCGATGAAGTGCAGAGCGGCGCGGGGCGTACCGGCACGCTGTTTGCGATGGAGCAGATGGGCGTTGCGCCGGATCTTACCACCTTTGCGAAATCGATCGCGGGCGGCTTCCCGCTGGCGGGCGTCACCGGGCGCGCGGAAGTAATGGATGCCGTCGCTCCAGGCGGTCTGGGCGGCACCTATGCGGGTAACCCGATTGCCTGCGTGGCTGCGCTGGAAGTGTTGAAGGTGTTTGAGCAGGAAAATCTGCTGCAAAAAGCCAACGATCTGGGGCAGAAGTTGAAAGACGGATTGCTGGCGATAGCCGAAAAACACCCGGAGATCGGCGACGTACGCGGGCTGGGGGCGATGATCGCCATTGAGCTGTTTGAAGACGGCGATCACAACAAGCCGGACGCCAAACTCACCGCCGAGATCGTGGCTCGCGCCCGCGATAAAGGCCTGATTCTTCTCTCCTGCGGCCCGTATTACAACGTGCTGCGCATCCTTGTACCGCTCACCATTGAAGACGCTCAGATCCGTCAGGGTCTGGAGATCATCAGCCAGTGTTTTGATGAGGCGAAGCAGTGA |
| **Spytag** | GGCTCAGGTGCACATATCGTCATGGTTGATGCGTACAAACCGACCAAAGGCAGCGGA |
| **EutM~SpyCatcher** | ATGGGCAGCAGCCATCACCATCATCACCACAGCCAGGATCCGAATTCGATGGCGGATGTGCAGATGATCGCGCTGGGCATGATTGAAACCAAGGGTCTGGTGGCGGCGATTGAAGCGGCGGATGCGATGGTGAAAGCGGCGAACGTTAAGCTGATCGGCAAAGAGTACATTGGTGGCGGTCTGGTGACCGTTATGGTTCGTGGCGACGTGGGTGCGGTTAAAGCGGCGACCGATGCGGGTGCTGCGGCGGCGCAGCGTATCGGCGAGCTGGTTAGCGTGCACGTTATTCCGCGTCCGCACGGTGATGCGGAAATGATTCTGCCGAGCGCGAAAGGTTCTGGTTCTGGTTCTGGTTCTGGTTCTGGTTCTGGCGGGAGCGCTATGGTGGACACCCTGTCGGGCCTCTCTAGTGAACAGGGGCAAAGCGGCGATATGACTATCGAAGAAGATTCAGCGACGCATATTAAGTTTTCGAAGCGCGATGAAGATGGGAAGGAACTCGCAGGAGCAACTATGGAGCTGCGTGACAGTAGCGGGAAGACAATTTCGACCTGGATTAGTGATGGCCAAGTTAAAGACTTTTATCTGTATCCAGGCAAATATACCTTCGTAGAAACCGCAGCACCTGATGGTTATGAAGTCGCGACCGCTATTACTTTTACAGTGAACGAGCAGGGGCAGGTCACCGTCAATGGGAAAGCTACGAAAGGGGATGCCCATATTGACTAA |
| **YahK** | ATGAAGATCAAAGCTGTTGGTGCATATTCCGCTAAACAACCACTTGAACCGATGGATATCACCCGGCGTGAACCGGGACCGAATGATGTCAAAATCGAAATCGCTTACTGTGGCGTTTGCCATTCCGATCTCCACCAGGTCCGTTCCGAGTGGGCGGGGACGGTTTACCCCTGCGTGCCGGGTCATGAAATTGTGGGGCGTGTGGTAGCCGTTGGTGATCAGGTAGAAAAATATGCGCCGGGCGATCTGGTCGGTGTCGGCTGCATTGTCGACAGTTGTAAACATTGCGAAGAGTGTGAAGACGGGTTGGAAAACTACTGTGATCACATGACCGGCACCTATAACTCGCCGACGCCGGACGAACCGGGCCATACTCTGGGCGGCTACTCACAACAGATCGTCGTTCATGAGCGATATGTTCTGCGTATTCGTCACCCGCAAGAGCAGCTGGCGGCGGTGGCTCCTTTGTTGTGTGCAGGGATCACCACGTATTCGCCGCTACGTCACTGGCAGGCCGGGCCGGGTAAAAAAGTGGGCGTGGTCGGCATCGGCGGTCTGGGACATATGGGGATTAAGCTGGCCCACGCGATGGGGGCACATGTGGTGGCATTTACCACTTCTGAGGCAAAACGCGAAGCGGCAAAAGCCCTGGGGGCCGATGAAGTTGTTAACTCACGCAATGCCGATGAGATGGCGGCTCATCTGAAGAGTTTCGATTTCATTTTGAATACAGTAGCTGCGCCACATAATCTCGACGATTTTACCACCTTGCTGAAGCGTGATGGCACCATGACGCTGGTTGGTGCGCCTGCGACACCGCATAAATCGCCGGAAGTTTTCAACCTGATCATGAAACGCCGTGCGATAGCCGGTTCTATGATTGGCGGCATTCCAGAAACTCAGGAGATGCTCGATTTTTGCGCCGAACATGGCATCGTGGCTGATATAGAGATGATTCGGGCCGATCAAATTAATGAAGCCTATGAGCGAATGCTGCGCGGTGATGTGAAATATCGTTTTGTTATCGATAATCGCACACTAACAGACTGA |
| **GdhA** | ATGGATCAGACATATTCTCTGGAGTCATTCCTCAACCATGTCCAAAAGCGCGACCCGAATCAAACCGAGTTCGCGCAAGCCGTTCGTGAAGTAATGACCACACTCTGGCCTTTTCTTGAACAAAATCCAAAATATCGCCAGATGTCATTACTGGAGCGTCTGGTTGAACCGGAGCGCGTGATCCAGTTTCGCGTGGTATGGGTTGATGATCGCAACCAGATACAGGTCAACCGTGCATGGCGTGTGCAGTTCAGCTCTGCCATCGGCCCGTACAAAGGCGGTATGCGCTTCCATCCGTCAGTTAACCTTTCCATTCTCAAATTCCTCGGCTTTGAACAAACCTTCAAAAATGCCCTGACTACTCTGCCGATGGGCGGTGGTAAAGGCGGCAGCGATTTCGATCCGAAAGGAAAAAGCGAAGGTGAAGTGATGCGTTTTTGCCAGGCGCTGATGACTGAACTGTATCGCCACCTGGGCGCGGATACCGACGTTCCGGCAGGTGATATCGGGGTTGGTGGTCGTGAAGTCGGCTTTATGGCGGGGATGATGAAAAAGCTCTCCAACAATACCGCCTGCGTCTTCACCGGTAAGGGCCTTTCATTTGGCGGCAGTCTTATTCGCCCGGAAGCTACCGGCTACGGTCTGGTTTATTTCACAGAAGCAATGCTAAAACGCCACGGTATGGGTTTTGAAGGGATGCGCGTTTCCGTTTCTGGCTCCGGCAACGTCGCCCAGTACGCTATCGAAAAAGCGATGGAATTTGGTGCTCGTGTGATCACTGCGTCAGACTCCAGCGGCACTGTAGTTGATGAAAGCGGATTCACGAAAGAGAAACTGGCACGTCTTATCGAAATCAAAGCCAGCCGCGATGGTCGAGTGGCAGATTACGCCAAAGAATTTGGTCTGGTCTATCTCGAAGGCCAACAGCCGTGGTCTCTACCGGTTGATATCGCCCTGCCTTGCGCCACCCAGAATGAACTGGATGTTGACGCCGCGCATCAGCTTATCGCTAATGGCGTTAAAGCCGTCGCCGAAGGGGCAAATATGCCGACCACCATCGAAGCGACTGAACTGTTCCAGCAGGCAGGCGTACTATTTGCACCGGGTAAAGCGGCTAATGCTGGTGGCGTCGCTACATCGGGCCTGGAAATGGCACAAAACGCTGCGCGCCTGGGCTGGAAAGCCGAGAAAGTTGACGCACGTTTGCATCACATCATGCTGGATATCCACCATGCCTGTGTTGAGCATGGTGGTGAAGGTGAGCAAACCAACTACGTGCAGGGCGCGAACATTGCCGGTTTTGTGAAGGTTGCCGATGCGATGCTGGCGCAGGGTGTGATTTAA |

**Table S2.** Primers used in this study.

| Name | Primer sequence (5’-3’) |
| --- | --- |
| *CadA*-F | GCCAGGATCCGAATTCATGAACGTTATTGCAATATTGAATCACATGG |
| *CadA*-R | CCTGCAGGCGCGCCGAGCTCAATACCTTAACGGTATAGCGGCC |
| *GabT*-F | AAGGAGATATACATATGATGAACAGCAATAAAGAGTTAATGCAGC |
| *GabT*-R | TATCCAATTGAGATCTCTGCTTCGCCTCATCAAAACACT |
| *YahK-*F | AGGAAACAGACCATGATGAAGATCAAAGCTGTTGGTGCA |
| *YahK*-R | CTAGAGGATCCCCGGTCAGTCTGTTAGTGTGCGATTATCGATAACAAAAC |
| linker-R | TGCACCTGAGCCGTCGACACCGGATCCCCCTGAACCCTGCTTCGCCTCATCAAAACACT |
| *Spytag*-R | TCCGCTGCCTTTGGTCGGTTTGTACGCATCAACCATGACGATATGTGCACCTGAGCCGTCG |
| *GabT~Spytag-R* | GGTTCAGGGGGATCCGGTGTCGAC |
| *EutM~SpyCatcher-F* | CGATCGCTGACGTCGGTACCAAGGAGATATACCATGGGCAGC |
| *EutM~SpyCatcher-R* | TTACCAGACTCGAGGGTACCTTAGTCAATATGGGCATCCCCTTTCG |
| *GdhA-F* | CCGGGGATCCTCTAGAGGGTTTTATATCTATGGATCAGACATATTCTCT |
| *GdhA-R* | CAAAACAGCCAAGCTTTTAAATCACACCCTGCGC |
